# Hyperglycemia and Venous Thromboembolism

**DOI:** 10.3390/diagnostics14171994

**Published:** 2024-09-09

**Authors:** Neha Panchagnula, William Philip Brasher

**Affiliations:** Section of Pulmonary, Critical Care, and Sleep Medicine, Department of Medicine, Baylor College of Medicine, Houston, TX 77030, USA

**Keywords:** hyperglycemia, diabetes mellitus, venous thromboembolism, pulmonary embolism, hypercoagulability

## Abstract

Patients with diabetes mellitus (DM) have chronically increased blood glucose and multiple physiologic alterations that place them at elevated risk for vascular disease. Traditionally, this vascular risk has mainly referred to chronic atherosclerosis and embolic arterial disease. Retrospective studies have suggested an increased risk of a pulmonary embolism (PE) and deep vein thrombosis (DVT), collectively termed venous thromboembolism (VTE), in patients with DM, but this association has been difficult to demonstrate with comorbidities such as obesity in meta-analysis. Clinical studies have demonstrated worse outcomes for patients with DM who suffer from VTE. In vitro studies show multiple physiologic abnormalities with chronic inflammation, endothelial dysfunction, dysfunction in the coagulation cascade, as well as other changes that drive a vicious cycle of hypercoagulability. Aggressive medical management of DM can improve vascular outcomes, and some anti-hyperglycemic therapies may modify VTE risk as well. Anticoagulation strategies are similar for patients with DM, but with some added considerations, such as high rates of comorbid renal dysfunction. More research is needed to definitively categorize DM as a risk factor for VTE and elucidate specific therapeutic strategies.

## 1. Introduction

Pulmonary embolism (PE) and deep vein thrombosis (DVT), collectively termed venous thromboembolism (VTE), are two of the most important causes of morbidity in the world, affecting over ten million people [1]. PE comprises clots that typically form in the large veins of the lower extremity that travel to the pulmonary vasculature. PE has a 90-day mortality of up to 20%, although this is at least partially attributed to comorbidities [2]. Major causes of VTE include cancer, surgery, and other pathologies associated with Virchow’s triad of endothelial dysfunction/injury, stasis, and hypercoagulability. While diabetes mellitus (DM) and hyperglycemia are known to cause arterial thrombosis and vascular disease, evidence suggests that they may play a role in an increased risk of VTE as an additional source of vascular disease. The management and research of DM has typically focused on the prevention of these microvascular and macrovascular arterial complications, as the potentially increased risk of VTE has been controversial. Hyperglycemia and insulin resistance affect platelet count and aggregation, the modification of coagulation factors, and thrombolysis in vitro [3]. Patients with DM have numerous comorbidities including obesity, hypertension, and inflammatory and hormonal complications, which are also risk factors for VTE and thus serve as cofounders when examining the role of DM as an independent risk factor. DM and acute hyperglycemia may also lead to poorer outcomes in those who develop VTE. In this review, we will explore the effect of acute and chronic hyperglycemia on the development of VTE, clinical outcomes, and potential therapies.

## 2. Clinical Links between Hyperglycemia and VTE

### 2.1. DM as an Indepdent Risk Factor

There have been links for multiple years between hyperglycemia and an increased risk for developing VTE, with conflicting data from observational studies [4,5]. Acute hyperglycemia in hospitalization and chronic hyperglycemia associated with DM are not always clearly distinguished, although it is plausible these have separate effects on risk and outcomes. Diabetes has been known to lead to significant atherosclerotic disease, and there has been speculation of a link between this and VTE [6]. Early retrospective studies and meta-analyses showed that DM may be a strong risk factor for VTE, but these did not control for known confounders of both diseases such as obesity [7]. DM is also a heterogeneous disease with patients with type 2 DM (T2DM) developing insulin resistance over years with different comorbidities, compared with type 1 DM (T1DM), which presents earlier in life in the setting of immune-mediated pancreatic beta cell destruction. Outcomes from VTE and PE may be affected by DM and acute hyperglycemia that we will also explore further. 

### 2.2. T2DM and VTE Risk

T2DM has been proposed as a significant risk factor for the development of VTE but is difficult to demonstrate conclusively due to multiple cofounders including obesity and a sedentary lifestyle, among other factors. A recent retrospective study involving a British cohort of patients with T2DM revealed a slight increase in risk of VTE by hemoglobin A1c level > 7.0%, but only in women with a A1c in the last 90 days with an OR 1.55 (95% CI: 1.08–2.24). There was no linear correlation between risk and higher A1c levels, and no overall correlation in the full population. The study did take in to account such confounders as BMI [8]. Another retrospective case–control from an Austrian cohort found that individuals with T2DM had 1.4 times higher risk of developing VTE compared to those without the condition OR 1.4 (95% CI: 1.36–1.43). There was a higher risk seen in females compared to males [9]. The latter was a hospital-based study and was able to correct for some risk factors such as obesity but not for contraception use. 

Although there have been several retrospective studies suggesting links between chronic hypoglycemia, major meta-analyses have not been positive. Several were performed from 2016 through 2023 with different inclusion criteria and failed to find a significant link between T2DM and VTE [10,11,12]. One meta-analysis that did find a positive examined patients with gestational DM and found a positive association RR 1.28 (95% CI: 1.13–1.46) [13]. Gestational DM is a disease that affects a unique patient population. Pregnancy itself is a prothrombotic condition, which may be modified by hyperglycemia. Overall, it appears, however, that type 2 DM is unlikely to be a significant independent risk factor for VTE. The likely main confounder addressed by meta-analyses is body mass index (BMI), which correlates closely with T2DM [14]. An elevated BMI is a classic cardiovascular risk factor correlating strongly with VTE and has multiple metabolic effects separate from T2DM, including circulating lipid levels, which could act as confounders [15,16].

### 2.3. T1DM and VTE Risk

Patients with T1DM lack insulin production, have fewer options for controlling insulin, and experience poorer cardiovascular outcomes than those with T2DM. T1DM patients are typically less obese and have different comorbidities. Autoimmunity underlies this condition, so patients may suffer from chronic inflammation separate from that seen in T2DM and have a different risk profile for VTE [17]. Few studies specifically examine T1DM or separate type 1 from type 2 when studying DM. One British study analyzed the conditions separately and found that only T2DM was associated with an increased risk of VTE with an HR of 1.46 (95% CI: 1.11–1.92), while T1DM was not with an HR of 1.06 (95% CI: 0.98–1.14) [18]. One large retrospective study looking at VTE specifically in patients with T1DM, after adjusting for comorbidities, established a strong association between T1DM and VTE with an adjustment HR of 5.33 (95% CI: 3.57–7.96) [19]. More studies are needed to assess this risk and delineate potential differences in thrombotic risk between T1DM and T2DM.

### 2.4. Hyperglycemia and VTE Outcomes

While an unclear risk factor for VTE formation, DM and hyperglycemia may also influence outcomes. In a single-center study, multivariate analysis revealed that for patients undergoing knee surgery, a preadmission blood glucose (BG) level of at least 200 mg/dL independently increased the risk of PE by 3.19 times (*p* = 0.015) compared to those with a BG level of less than 110 mg/dL [20]. In patients with acute PE, elevated admission BG is a common finding and has been independently associated with increased short-term mortality [21]. This observation underscores the critical impact of glycemic control on the prognosis of acute PE [21]. An observational study of around 500 patients demonstrated that even in patients without a prior diagnosis of DM, markedly elevated arterial BG levels upon hospitalization for VTE correlate with higher mortality rates. The paper defined an arterial BG of 111–140 as mildly elevated, 141–180 mg/dL as moderately elevated, and >180 mg/dL as markedly elevated, and found hazard ratios of 1, 6, 2.3, and 4.7 for increased mortality, respectively, with increasing BG [22]. Even more transient stress hyperglycemia in a small study was associated with a larger size of PE, more proximal location, and higher severity index per PESI (Pulmonary Embolism Severity Index), a commonly used risk calculator to determine expected mortality and long-term morbidity based on a variety of PE risk factors [23].

DM has also been linked to increased hospitalization and mortality in patients who develop PE. [24] A large study of almost 1.2 million PE patients in Germany found increased in-hospital mortality, even when adjusted for age and comorbidities (OR 1.21, 95% CI: 1.20–1.23). In this cohort, there was increased bleeding, the use of thrombolytics, and shock in patients with DM [24]. Another large study based on a Spanish national database suggested an increased diagnosis of PE for those with DM and in-hospital mortality for men with OR 1.22 (95% CI: 1.12–1.32) and women with a OR of 1.24 (95% CI: 1.15–1.33) [25]. Diez and associates from the same group later conducted a multinational matched cohort study based on a VTE registry and found that patients with DM undergoing anticoagulation therapy exhibited higher mortality rates compared to their non-diabetic counterparts with a HR of 1.45 (95% CI: 1.25–1.67). This was at least in part due to other arterial ischemic events such as stroke, with DM not identified as an independent risk factor for mortality with PE [26]. This increased mortality in diabetic patients suggests that the presence of DM exacerbates the severity and the complications associated with PE, although these are, again, potentially impacted by comorbidities. The interplay between hyperglycemia, a pro-inflammatory state, and the hypercoagulable conditions in DM likely contribute to poorer clinical outcomes overall. Therefore, managing DM with tighter glycemic control could be vital in reducing mortality and improving overall prognosis in PE patients.

Post-thrombotic disease could also be affected by DM. Using a large data set of patients with acute PE, several risk factors were identified, and a claims-based risk model has been developed to predict the risk of chronic thromboembolic pulmonary hypertension (CTEPH) following a PE event. DM was one factor identified as causing an increased risk of CTEPH following PE (OR 1.07, 95% CI: 1.02–1.11) [27]. This suggests that PE patients with DM need to be followed closely after the event to make sure that they do not develop CTEPH, which can be a lethal condition. Interestingly, in a smaller case–control study comparing CTEPH patients with those with idiopathic pulmonary hypertension (IPAH), DM was more strongly associated with IPAH [28]. These results do not imply however that DM does not increase the risk of CTEPH in the general population, just that it appeared to be more strongly associated with IPAH in the cohort. Clearly more research is needed to separate confounders, but current evidence suggests that DM and hyperglycemia affect and possibly lead to worse outcomes with VTE in terms of mortality and long-term complications (Table 1).

## 3. Proposed Mechanisms between Hyperglycemia and Hypercoagulability

### 3.1. ROS, Inflammation and Hyperglycemia

While the increased clinical risk is unclear for VTE in the setting of hyperglycemia, the in vitro data of prothrombotic risk are stronger through a variety of mechanisms including hyperinflammatory states, endothelial activation, the alteration of the clotting cascade, platelet interactions, and impaired fibrinolysis. In hyperglycemic states, the interaction between advanced glycation end products (AGEs) and their receptor, RAGE (Receptor for AGEs), plays a critical role in the development of chronic inflammation and hypercoagulability. Elevated levels of BG lead to the formation of AGEs, which are modified proteins or lipids resulting from non-enzymatic reactions with sugars. These AGEs engage with RAGE, triggering a cascade of intracellular events that contribute to chronic inflammation and oxidative stress. In vitro studies have linked RAGE activation to vascular inflammation and injury, both of which promote clot activation [30]. RAGE inhibition also led to reduced lung damage in acute respiratory distress syndrome, and activation perhaps promotes hypercoagulability by allowing the release of coagulation factors through this vascular permeability [31]. The AGE–RAGE interaction not only promotes inflammation directly, but also exacerbates the diabetic milieu by inducing the production of reactive oxygen species (ROS). Elevated glucose levels have been reported to fuel ROS-mediated NF-κB (nuclear factor kappa B) activation, amplifying the expression of RAGE [32]. The intricate interplay between AGEs and RAGE contributes to a cascade of events linking high glucose concentrations to ROS production and subsequent neutrophil-related cell death (NETosis), which releases neutrophil extracellular traps (NET) [33]. NETs are complex structures comprised of decondensed chromatin and granules containing degradative enzymes [34]. While the benefits of NETosis in combating infections are well-established, recent research has uncovered its detrimental effects in autoimmune diseases and diabetes. In the context of diabetes, characterized by chronic low-grade inflammation, NETosis is activated by pro-inflammatory cytokines and ROS [33]. NETosis’s main function is to clear pathogens, but it also appears to play a clear role in thrombus formation through the activation of the thrombus and acting as a scaffold for fibrin formation [35]. The AGE–RAGE axis serves as a key link between hyperglycemia, inflammation, and the development of the thrombus, highlighting its significance as a potential therapeutic target in managing diabetes-related complications. Hyperglycemia induces mitochondrial damage, collateral glucose routes, and spontaneous glucose reactions. All processes promote excessive ROS generation leading to oxidative stress. Looking in the opposite direction, oxidative stress induces the AGE–RAGE pathway, which then further stimulates mitochondrial impairment, glucose collateral routes, and insulin resistance, leading to the reinforcement of hyperglycemia [36]. ROS are directly linked to both increased thrombus formation and decreased resolution by a variety of mechanisms, providing an additional plausible mechanism for the AGE–RAGE axis to lead downstream to hypercoagulability [37].

### 3.2. Hypofibrinolysis and Clot Strength in Hyperglycemia

**Figure 1:**
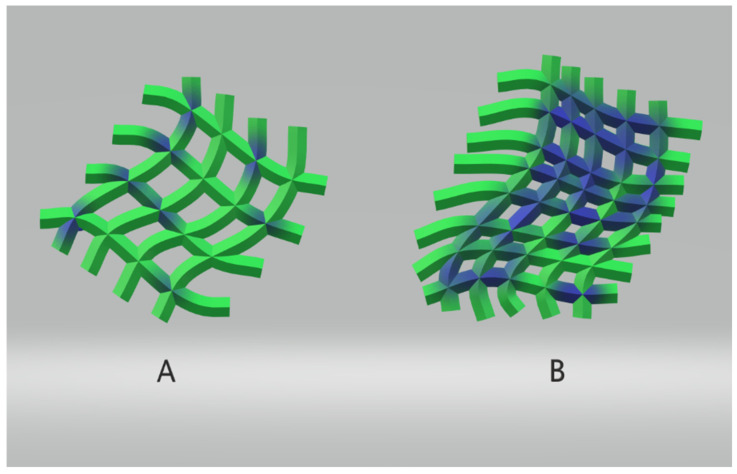
Schematic of influence of glucose concentration on clot strength. To the left, (**A**) demonstrates a more delicate fibrin strand network (in green) with lower glucose concentration (in blue); to the right, (**B**) demonstrates a denser fibrin strand network (green) resistant to fibrinolysis in the presence of higher glucose concentrations (blue) [38].

Individuals with DM form compact fibrin networks that are resistant to fibrinolysis and have a reduced efficacy in the fibrinolytic system [38]. The two most critical inhibitors in fibrinolysis are plasminogen activator inhibitor 1 (PAI-1) and alpha-2 plasmin inhibitor (α2PI, or α2-antiplasmin) [39]. The profound suppression of fibrinolysis in T2DM is primarily mediated by increased levels of PAI-1 [40]. Research shows that elevated concentrations of fibrinogen and thrombin in DM accelerate the formation of fibrin clots and contribute to thrombi production with increased fiber density. The highest levels of fibrinogen glycation were observed under hyperglycemic conditions [41]. The coagulation cascade, activated through various pathways, culminates in the formation of a stable fibrin clot. This stability is augmented by covalent cross-links between fibrin molecules catalyzed by factor XIIIa [42]. The compromised fibrinolysis, coupled with elevated PAI-1, underscores the hypofibrinolytic characteristic of hyperglycemic conditions, influencing the strength and stability of blood clots (Figure 1).

### 3.3. Coagulation Cascade and Hyperglycemia

Hyperglycemia and hyperinsulinemia in DM affect the intrinsic coagulation pathway, with studies revealing increased factor VIII, IX, and XI levels per mmol/L rise in fasting plasma glucose [43,44]. The Netherlands’ Epidemiology of Obesity study demonstrated that these associations remained significant after adjusting for confounding factors such as age, gender, and BMI, among others. Patients with impaired insulin sensitivity exhibited heightened synthesis of factor XII, XI, and IX in hepatocytes, coupled with a shorter activated partial thromboplastin time, possibly mediated by insulin resistance-induced low-grade inflammation [45]. Other studies have also shown differences in the quality and quantity of coagulation factors in diabetic patients [46].

**Figure 2:**
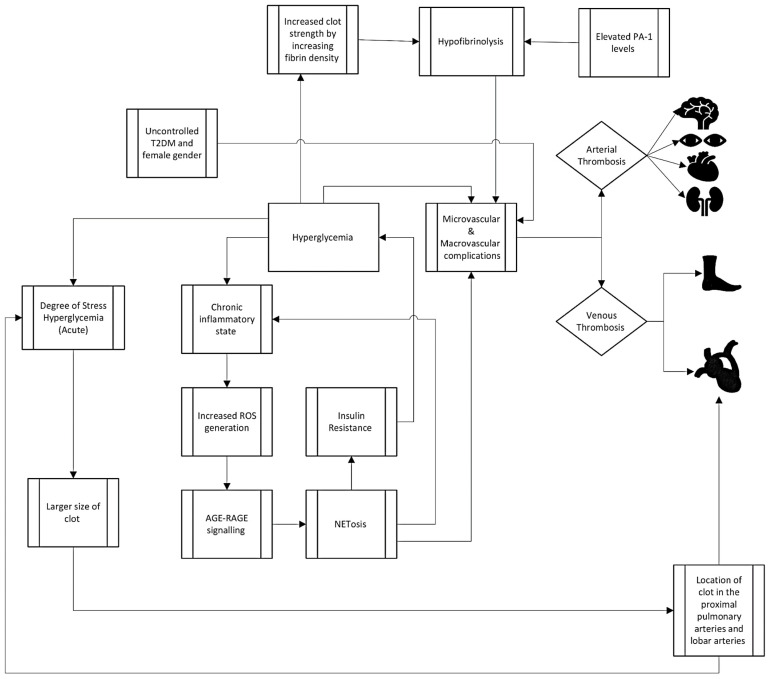
Summary of proposed mechanisms of clot formation in hyperglycemia.

A chronic hyperglycemic state leads to high circulating levels of thrombin–antithrombin complexes (TAT), increased tissue factor procoagulant activity (TF PCA), and factor VII [47]. TF converts Factor VII to the activated form of VIIA. With rising levels of BG and insulin, TF-PCA also proportionally increases. However, when attempting to reduce the glucose levels rapidly, TAT levels and TF PCA activity do not appear to reduce significantly [48]. In this model, insulin and elevated BG together may play a role in the activation of the coagulation cascade (Figure 2).

### 3.4. Endothelial Dysfunction in Hyperglycemia

Endothelial dysfunction induced by diabetes has a well-established role in arterial complications in the microvasculature with reduced NO production and availability, alterations in VEGF, and high levels of oxidative stress all leading to poor perfusion and increased thrombosis [49]. However, the direct link between this dysfunction and VTE in DM clinically has not been clearly demonstrated, but multiple abnormalities of endothelial dysfunction have been demonstrated in long term in patients who suffer from VTE [50]. The exposure of the vascular endothelium to higher glucose concentrations seems to be an important precipitant of endothelial dysfunction. The glycocalyx, a protective layer of proteoglycans on the endothelium, plays a crucial role in maintaining vessel wall integrity. Elevated levels of hyperglycemia and hyperinsulinemia were shown to potentially disrupt the glycocalyx, contributing to vascular dysfunction during periods of heightened glucose levels [51,52].

**Figure 3:**
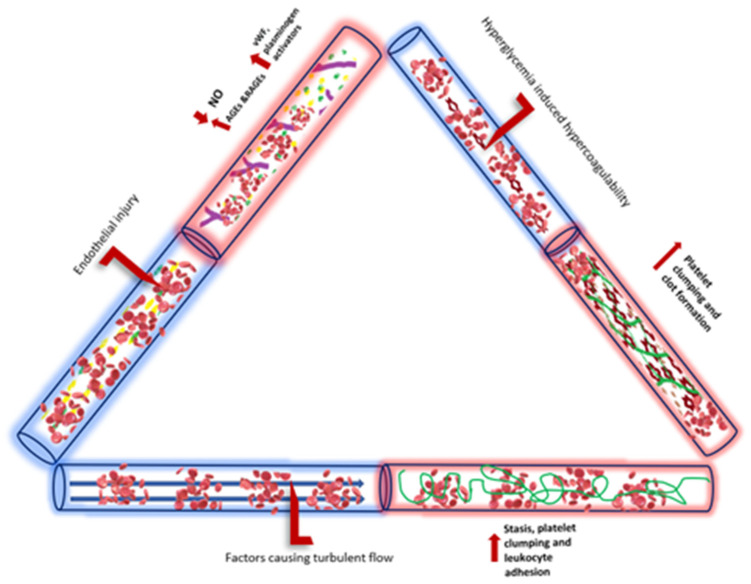
Summary of hyperglycemic effects on Virchow’s triad of endothelial injury, blood flow, and hypercoagulability.

Both cardiovascular disease and type 2 diabetes exhibit an inadequate regulation of the endothelial cell redox environment, marked by an imbalance that leans towards the excessive production of reactive oxygen species (ROS) by NADPH oxidase (NOX) [53]. Investigations in type 2 diabetes models reveal that this abnormal activation of NOX plays a significant role in the decoupling of endothelial nitric oxide synthase (eNOS), contributing to endothelial dysfunction. Given that endothelial dysfunction is a well-established precursor to cardiovascular disease, NOX emerges as crucial molecular connectors linking type 2 diabetes to the development of vascular complications 53 (Figure 3). 

## 4. Treatment Implications VTE with Hyperglycemia

### 4.1. Effect of Anti-Diabetic Drugs on VTE

Various drugs used to treat diabetes may have differing effects on the modification of VTE risk, either by improving glycemic control or through having antithrombotic properties. Metformin has been found to have a variety of effects outside of glycemic control, with a meta-analysis showing decreased all-cause mortality and fewer cardiovascular events, compared to other drugs like sulfonylureas [54]. One systematic review, encompassing observational studies, found a decreased rate of VTE with metformin, with a relative risk reduction of 22 to 58% associated with use of the drug [55]. Several antithrombotic mechanisms have been demonstrated with metformin use, including decreased platelet function and activation, a reduction in C reactive protein, decreased Factor VII, and others [56]. In both normal and diabetic rat models, metformin has been shown to decrease both the size and formation of arterial and venous thrombi, as well as the incidence of PE [47].

Glucagon-like petide-1 receptor agonist (GLP-1 RA) drugs mimic endogenous hormones released by the gastrointestinal (GI) tract and delay gastric emptying, upregulate glucose, and downregulate glucagon while increasing insulin response. Several GLP-1 RAs have become FDA-approved and are popular due to the significant reduction in HbA1c observed in addition to weight loss. The main side effects reported in trials are GI in nature [57]. Evidence from a meta-analysis including several clinical trials supports improved cardiovascular outcomes and decreased stroke risk with these drugs [58]. GLP-1 RAs have consistently shown a reduction in atherothrombotic events, all-cause cardiac mortality, and the progression of CKD, thus leading to the American Heart Association and other societies recommending their use [59]. GLP-1 RA therapy does appear to reduce multiple markers of innate inflammation, in addition to atherosclerotic plaque such as Il-6 and TNF-α, which may decrease VTE risk [60,61].

Sodium-glucose cotransporter 2 inhibitors (SGL2) increase glucose excretion in the kidney and have also consistently shown improved cardiovascular and renal outcomes, similar to GLP-1 RAs. There was concern that increases in hematocrit may increase blood viscosity and lead to more VTE. However, a meta-analysis suggests no association between SGL2 and VTE—there was no increased incidence of VTE, but there was also no beneficial effect seen on VTE reduction [62]. Robust cardiac outcomes have been impressive for both GLP-1 RA and SGL2 drugs, even though they may not have a direct effect on VTE risk or outcomes.

Even a lifestyle intervention involving weight loss over one year was shown to improve the coagulation profile, including a reduction in levels of protein C and S, and an increase in PTT [63]. In a small, randomized trial of coagulation factors after gastric surgery, all patients had increased fibrinolysis and reduced thrombin generation, but those who underwent a fitness regimen post-operatively had further increased fibrinolytic activity [64]. It is unclear if the mechanism by which this intervention works is by generating weight loss and reducing the thrombotic risk associated with obesity, or by improving glycemic control to minimize any excess risk attributable to DM.

### 4.2. Anticoagulation in Patients with VTE and Hyperglycemia

An increased risk of bleeding has been identified in patients with DM undergoing anticoagulation. In a Chinese study of 536 patients followed prospectively, DM was identified as an independent risk factor for major bleeding (OR 2.11, 95% CI: 1.10–4.12) with patients with clinically relevant bleeding having a higher HbA1C [65]. A German study also identified increased GI and intracerebral bleeding in patients with DM [24]. In Piazza et al., 2488 VTE patients from the USA were studied and found to have an increased risk of long-term bleeding complications in those with DM. An increased use of aspirin therapy (OR 1.59, 95% CI: 1.1–2.3) and chronic kidney disease (OR 2.19, 95% CI: 1.44–3.35), along with multiple other comorbidities, was seen in patients with DM, which could explain this increased bleeding risk [66]. Symptomatic atherosclerosis, which is higher in patients with DM, has been directly linked with higher bleeding risk in patients with PE [67]. Besides antiplatelet therapy, patients with DM are often at risk for other arterial diseases such as coronary artery disease or stroke, and for vascular malformations related to chronic hyperglycemia, which could also increase risk of bleeding [68].

There are no special guidelines regarding anticoagulation for prophylaxis or treatment in patients with DM, despite these elevated risks of bleeding. Evidence from the literature for atrial fibrillation trials with direct oral anticoagulants (DOAC) compared to vitamin K antagonists (VKA) suggests that DOAC are superior in both the primary outcomes of reduced risk of stroke and major intracranial bleeding [69]. Further data obtained after the initiation of DOAC showed reduced vascular complications and decreased bleeding in patients with type 2 DM compared to VKA [70]. In addition, there is concern for worsening renal function and coronary calcification with the use of VKA in the diabetic population. VKA drugs are even less desirable, especially with fluctuations in levels based on dietary intake, warranting strict dietary restrictions and the continued close monitoring of the INR [71]. Although DOAC therapy is likely superior to VKAs, monitoring renal function is still extremely important in patients with DM due to the high prevalence of chronic kidney disease and the partial renal excretion of these DOACs [72].

## 5. Conclusions

The definitive link between hyperglycemia, DM, and VTE with PE remains inconclusive. Recent evidence from in vitro studies supports cellular level disturbances from hyperglycemia that increase the risk of a prothrombotic state, which may provide biologic plausibility for a link with VTE. However, clinical implications of these in vitro effects of hyperglycemia on VTE/PE remain unclear. Clinically, elevated glucose levels increase mortality and worsen short-term outcomes in affected patients with VTE. Yet, numerous retrospective studies and meta-analyses have failed to demonstrate that T1DM, T2DM, or uncontrolled hyperglycemia represent independent risk factors for the development of VTE. In addition, the role of anti-glycemic therapy or special anticoagulation strategies in these patients is an important area of uncertainty that warrants further research. The further elucidation of the underlying mechanisms of VTE and hyperglycemia, and the development of novel therapies to address these mechanisms, is necessary to better care for patients with VTE and hyperglycemia and develop risk reduction strategies to improve clinical outcomes in the future.

## Figures and Tables

**Table 1:** Summary of major studies examining the association of DM and hyperglycemia with risk of developing VTE and risk of adverse outcome.

Retrospective Studies	Study Type	Country; Year(s)	Number of Patients	Results
Peng et al., 2020 [19]	Cohort	Taiwan; 2003–2011	24,835	Positive association between T1DM and VTE: HR 5.33 (95% CI 3.57–5.87)
Charlier et al., 2022 [8]	Case– Control	Switzerland, USA; 1995–2019	2653 VTE cases, 10,612 controls	No association in men between VTE and Hemoglobin bA1c Female T2DM patients with HbA1c levels > 8% slight increased risk OR 1.29 (95% CI 1.02–1.63)
Deischinger et al., 2022 [9]	Case– Control	Austria; 1997–2014	180,034 DM cases, 540,102 matched non-DM controls	Positive association between DM and VTE OR 1.4 (95% CI 1.36–1.43)
**Meta-Analysis**	**# of Studies Included**	**Country**	**Number of Patients**	**Results**
Gariani et al., 2015 [11]	24 studies	Multiple	~1.3 million	No association between DM and VTE. Adjusted HR 1.1 (95% CI 0.77–1.56);
Bai et al., 2015 [29]	15 studies	Multiple	803 million	Positive association between DM and VTE. HR 1.35 (95% CI 1.17–1.55)
Bell et al., 2016 [10]	19 studies	Multiple	~300,000	No association between DM and VTE: RR 1.1 (95% CI 0.94–1.29)
Xie et al., 2022 [13]	15 studies	Multiple	~8 million pregnant women	Positive association between gestational DM and VTE: RR 1.28 (95% CI 1.13–1.46)
Ding et al., 2023 [12]	50 studies	Multiple	5.8 million	No association between DM and VTE when adjusted for BMI: OR 1.04 (95% CI 0.94–1.15). DM patients with VTE associated with worse mortality OR 1.58 (95% CI 1.26–1.99)
**Retrospective Studies of Outcomes of DM Patients with VTE**	**Study Type**	**Country**	**Number of Patients**	**Results**
Schmitt et al., 2022 [24]	Case–Control	Germany	1.1 million	Positive association between DM and mortality with PE: Adjusted OR 1.21 (95% CI 1.20–1.23)
Akirov et al., 2016 [22]	Cohort	Israel	567	Positive association between hyperglycemia and increased mortality with PE: HR 2.3 (95% CI 1.2–4.5)
de Miguel-Díez et al., 2019 [26]	Cohort	Spain	6027	No association between DM and mortality with PE when adjusted for comorbidities: HR 1.26 (95% CI 0.97–1.63)
Kanwar et al., 2021 [27]	Case–Control	Canada	93,428	Positive association between DM and development of CTEPH: OR 1.07 (95% CI 1.02–1.11)
Scherz et al., 2012 [21]	Case–Control	USA	13,621	Positive association between elevated admission BG and increased mortality with PE: BG > 240 mg/dL OR 1.6 (95% CI 1.26–2.03)

HR-Hazard Ratio, OR-Odds Ratio, RR-Relative Risk, CI-Confidence Interval.
